# Cognitive functioning in patients with classical galactosemia: a systematic review

**DOI:** 10.1186/s13023-019-1215-1

**Published:** 2019-10-18

**Authors:** Merel E. Hermans, Mendy M. Welsink-Karssies, Annet M. Bosch, Kim J. Oostrom, Gert J. Geurtsen

**Affiliations:** 1 Department of Medical Psychology, Amsterdam UMC – location AMC, P.O. Box 22660, 1100 DD, Meibergdreef 9, 1105 AZ Amsterdam, The Netherlands; 2 Department of Pediatrics, Amsterdam UMC – location AMC, Meibergdreef 9, 1105 AZ Amsterdam, The Netherlands; 3 Psychosocial Department, Emma Children’s Hospital/Amsterdam UMC – location AMC, Meibergdreef 9, 1105 AZ Amsterdam, The Netherlands

**Keywords:** Classic Galactosemia, GALT deficiency, Neuropsychology, Neuropsychological functioning, Cognition, Critical review

## Abstract

**Background:**

Patients with the metabolic disorder classical galactosemia suffer from long-term complications despite a galactose-restricted diet, including a below average intelligence level. The aim of the current review was to investigate the incidence and profile of cognitive impairments in patients with classical galactosemia.

**Method:**

MEDLINE, EMBASE and PsychINFO were searched up to 23 October 2018 for studies examining information processing speed, attention, memory, language, visuospatial functioning, executive functioning and social cognition in patients with confirmed classical galactosemia utilizing standardized neuropsychological tests. Data synthesis followed a narrative approach, since the planned meta-analysis was not possible due to large variability between the neuropsychological assessments.

**Results:**

Eleven studies were included, including case-studies. The quality of most studies was moderate to low. As a group, patients with classical galactosemia exhibit below average to low scores on all cognitive domains. A large proportion of the patients perform on an impaired level on attention, memory and vocabulary. Evidence for impairments in information processing speed, language, visuospatial functioning and aspects of executive functioning was limited due to the small number of studies investigating these cognitive functions. Social cognition was not examined at all.

**Conclusions:**

Given the moderate to low quality of the included studies and the limited evidence in many cognitive domains, the incidence of cognitive impairment in patients with classical galactosemia is not yet clear. Both clinicians and researchers encountering patients with classical galactosemia need to be aware of possible cognitive impairments. Future well-designed studies are needed to determine the cognitive profile of classical galactosemia. This can be the basis for the development of intervention strategies.

## Introduction

Classical galactosemia (CG; *OMIM:* 230400) is a rare autosomal recessive metabolic disorder with an incidence between 1:16.000 and 1:60.000 in Europe and the USA [1]. The disorder is caused by a deficiency of galactose-1-phosphate uridylyltransferase (GALT, EC 2.7.7.12). Due to this deficiency, newborns develop a life-threatening illness after the ingestion of breast milk or formula. If CG is suspected, a galactose-restricted diet is started which eliminates the acute clinical symptoms [2]. CG is confirmed by means of absent or barely detectable GALT enzyme activity in red blood cells and/or the presence of two pathogenic *GALT* mutations [3]. Despite the lifelong diet, patients with CG develop long-term complications in different degrees of severity. Besides neurological deficits and primary ovarian insufficiency [1], cognitive functioning seems to be affected. A recent meta-analysis of intellectual functioning in a sample of early-treated patients with a confirmed CG-diagnosis found a mean below average total intelligence quotient (IQ) of 87. Only 15% had an average or above average IQ (100 or higher) in contradiction to the 50% found in the general population [4]. The lower level of cognitive functioning seems to affect the health-related quality of life [5] and a large proportion of the patients need additional care and guidance in the domain of mental functions [6]. Unfortunately, the majority of articles investigating the cognitive complications of CG only used intelligence tests which are designed to assess overall cognitive functioning [7] and not specific cognitive domains (i.e. information processing speed, executive functioning, memory, language, visuospatial functioning and social cognition). A low IQ can be caused by a global impairment in the general mental abilities, but it can also be the result of a specific impairment in one or multiple cognitive domains lowering scores of some or several subtests and consequently the IQ [8]. Consequently, it is important to delineate the cognitive profile of CG to understand the process underlying the lower level of cognitive functioning, to improve prognostic accuracy and to identify cognitive areas in which additional guidance and/or rehabilitative interventions are needed. Therefore, the aim of the current review is to systematically investigate cognitive functioning in patients with CG in order to answer the following questions:

1. What is the incidence of cognitive impairment in classical galactosemia?
2. Which cognitive domains are impaired in patients with classical galactosemia?

## Method

The current systematic review was performed according to the Preferred Reporting Items for Systematic Reviews and Meta-Analyses (PRISMA) method [9]. The PRISMA checklist of this review can be found in Additional file 1.

### Search strategy

The electronic databases MEDLINE, EMBASE and PsychINFO were searched up to 23 October 2018 with a medical information specialist. The search strategies for MEDLINE and EMBASE were developed to target the patient population and were modified by manually omitting irrelevant clusters of related articles identified by VOSviewer (see Additional file 2 [10];). The final search strategies are included in Additional file 3. In addition, reference lists of included articles and (systematic) reviews were hand searched. All records were de-duplicated in EndNote and all unique results were uploaded to the systematic review software program Covidence.

### Eligibility criteria

Both the title- and abstract screening and the subsequent full-text screening were independently performed by MEH and MMWK. Disagreement was resolved by consensus and consultation of GJG or AMB. Studies were included if they investigated patients with CG, confirmed by either genetic analysis with two pathogenic mutations and/or absent or barely detectable red blood cell GALT enzyme activity. Studies that selected a specific subgroup of CG patients based on clinical outcome were excluded. Moreover, studies needed to report standardized results of standardized neuropsychological tests examining cognitive domains. A standardized neuropsychological test requires standardized administration and scoring procedures, and the presence of normative data [8]. Studies solely assessing general cognitive status or intelligence were excluded. Studies only reporting aggregated scores of test batteries were also excluded.

Given the expected relatively small number of studies, there were no restrictions of age. Full-text, original articles of any publication year written in either English or Dutch were included. If multiple studies reported on the same patient cohort, the study reporting on the largest proportion of the cohort was included.

### Data extraction

Data on study characteristics (i.e. sample size, study design, control characteristics), patient characteristics (i.e. age, gender, criteria for diagnosis, age of diagnosis, age of start diet, compliance to diet, genetic mutation, clinical outcome and psychiatric symptoms) and cognitive outcomes were extracted from the included studies by both MEH and MMWK independently. Age of diagnosis and start of diet were included in the data extraction since late initiation of the galactose-restricted diet (i.e. after eight weeks) has been found to be related to lower intelligence levels in CG-patients [11, 12]. Moreover, the specific genetic mutations reported in the studies were extracted since some pathogenic mutations (i.e. S135 L) are associated with a less severe clinical outcome [13]. Clinical outcome was extracted since several outcomes might influence the performance on neuropsychological tests, including severe mental retardation, speech disorders and motor impairments [8] as well as psychiatric symptoms, especially mood disorders [14].

Four authors were contacted for numerical data that was solely described in their papers. One author responded within the set period of 1.5 months [15]. All neuropsychological (sub-)tests were categorized into different cognitive domains (i.e. information processing speed, attention, memory, language, visuoconstruction and visuospatial functioning, executive functioning and social cognition), based on neuropsychological handbooks [8, 16]. In the current review, test scores below the ninth percentile (i.e. z-score ≤ − 1.4) are described as impaired. A cognitive domain is defined as impaired if the results of multiple tests categorized in that particular cognitive domain are below the ninth percentile. For longitudinal designs, only the data of the first measurement was included.

### Risk of bias assessment

Risk of bias assessment was performed by MEH and verified by GJG. The Scottish Intercollegiate Guidelines Network (SIGN) quality appraisal checklists [17] were used for case-control studies. The critical appraisal checklists of the Joanna Briggs Institute (JBI [18]) were used for the remaining study designs. The assessment was done while taking the low incidence of the disease into account.

### Data synthesis

The outcome measures and quality of report of the results of all included studies were evaluated first. If multiple articles examined the same cognitive function with relatively similar tests and the quality of the studies was sufficient, a meta-analytic approach was planned by means of a random-effects model because of the expected heterogeneity between studies. If a quantitative approach was not feasible because of above-mentioned reasons, a narrative approach was planned.

## Results

### Study selection

**Fig. 1:**
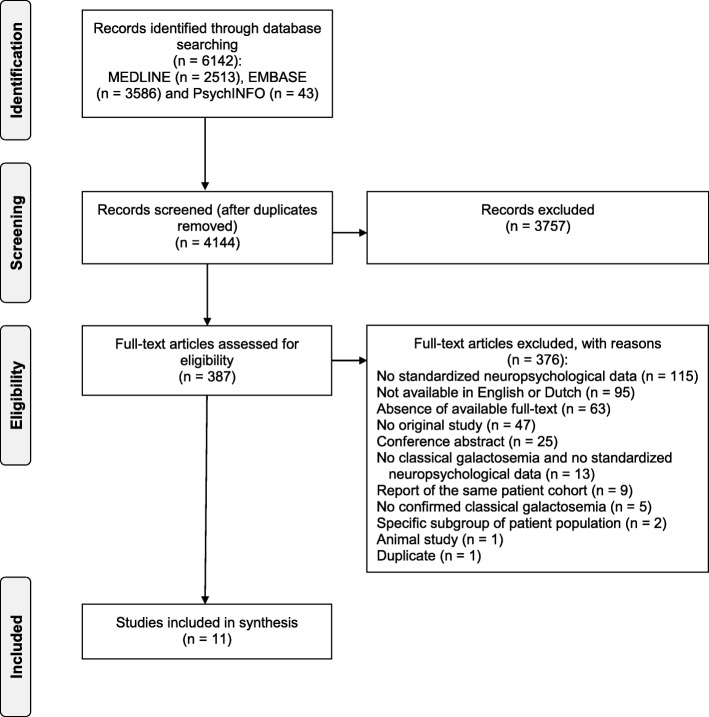
PRISMA flow diagram. Flow diagram of the study selection process of the current systematic review regarding cognitive functioning in patients with CG [9]

The database-search yielded a total of 6142 records. After the removal of duplicates, the remaining 4144 records were screened. Hand-searching the reference lists resulted in no extra articles. Finally, eleven studies were retained (see Fig. 1), including two case-control studies [19, 20], five cross-sectional studies [15, 21–24], one case-series [25] and three case reports [26–28]. Several studies investigating the cognitive development of patients with CG were excluded, since they only used developmental scales and/or intelligence tests which are not informative about the level of functioning in a specific cognitive domain.

### Study characteristics

The sample consisted of 177 patients with CG (see Table 1). The study sample sizes varied from one to 45. Six studies included only children, and five studies included both children and adults with an age range between two and 53 years. The genetic mutation was known of 71 patients, of which 48 were homozygous for the Q188R-variant might be related to a more severe outcome [1]. Due to the inclusion of four studies that did not perform or report the results of genetic analysis [21–23, 26], the presence of patients carrying the S135 L-variant remained unknown. Two studies reported the presence of movement disorders, in which tremor and ataxia were the most common symptoms [15, 23]. Psychiatric symptoms were present in two studies [15, 19] and lower intellectual functioning in the majority of the studies. All studies used normative data to evaluate cognitive functioning. Two studies additionally used control subjects [19, 20]. Both these studies matched their controls on age and gender, and one of them added parental educational coding as a matching variable [20]. Of one study, individual patient data were available making it possible to exclude six individual patients that received the diagnosis CG after 56 days (i.e. 8 weeks) in order to avoid any influence of late treatment [15].

**Table 1:** Study characteristics of the included studies

Author (year)	Study design	N [P]/[C]	Mean age y:mo (range)	Gender	Criteria for diagnosis	Age of diagnosis /start of diet	Genetic mutation (N)	Clinical outcome (N)	Psychiatric symptoms (N)
Antshel et al. (2004) [19]	Case Control	25 / 20	10:9 (8–14)	10 females, 15 males	Confirmed by genetic analysis	NR / first week of life	Q188R/Q188R (25)	Special education (14) IQ: 84.3 (*SD* = 8.3)	CDI: *z* = 0.14 (*SD =* 0.04) ADHD (4): no medication 24 h before NPA
Doyle et al. (2010) [21]	Cross-sectional	28	29:3 (15–53)	20 females, 8 males	Confirmed by very low GALT-activity in leucocytes	Neonatally / NR^a^	NI	IQ: 88.9 (*SD* = 16.8)	No psychiatric disorders.
Hoffmann et al. (2011) [22]	Cross-sectional	32	21:2 (9:9–37:4)	12 females, 20 males	Confirmed by GALT activity < 2% and genetic analysis	NR / NR	NR	IQ: 76.2 (*SD* = 14.8)	NR
Iakovou et al. (2018) [26]	Case Report	1	4	1 male	Confirmed by absent GALT activity	Neonatally/ Neonatally	NI	IQ: 115 (age of 19)	NR
Kaufman et al. (1995) [23]	Cross-sectional	45	4:0–39	22 females, 23 males	Confirmed by absence of GALT activity	Neonatally or in infancy / Neonatally or in infancy	NI	Tremor, ataxia and dysmetria (12)	NR
Lewis et al. (2012) [25]	Case series	4	7:77:88:59:2	3 females, 1 male	Confirmed by GALT activity < 1.3 units/ml blood and genetic analysis	5 days / 5 days (3) or birth (1)	Q188R/Q188R (4)	IQ: 118 IQ: 67 IQ: 60IQ: 73	NR
Lewis et al. (2013) [20]	Case Control	1 / 3	5:7	1 male	Confirmed by genetic analysis	6 days / 6 days	Q188R/Q188R	IQ: within average range	NR
Lewis et al. (2013) [27]	Case Report	1	2:1	1 female	Confirmed by GALT activity < 0.01 U/g Hb and genetic analysis	7 days / 7 days	Q188R/Q188R	Learning disabled IQ = 60	NR
Ng et al. (2003) [28]	Case Report	1	8	1 female	Confirmed by GALT activity (0.1–0.3%) and genetic analysis	± 2–3 weeks / ± 2–3 weeks	Q133R/other	IQ: 123 Normal ovarian function later in life	NR
Van Erven et al. (2017) [24]	Cross-sectional	12	17:4 (14:6–21:1)	9 females, 3 males	Confirmed by GALT activity (0.55%) and/or genetic analysis.	NR / Mean of 11 days (range: 0–35)	Q188R/Q188R (5) Q188R/other (1) L195P/K229 N (3) W134 fs/unknown (2) Unknown (1)	Special education (9)	NR
Waisbren et al. (2012) [15]	Cross-sectional	27	29:10 (18–51)	13 females, 14 males	Confirmed by absence of GALT activity and genetic analysis.	Mean of 12 days (range: 1–42)/NR	Q188R/Q188R (12) Q188R/other (12) K285 N/L195P (1) K127Q/deletion (1) Deletion/deletion (1)	IQ: 89.4 (*SD* = 19.5) Tremor (11) Ataxia (3) Dysarthria (6) Apraxia (2)	Depression (10) Anxiety (20)

*Notes. N* sample size, *P* patient group, *C* control group, *NBS* Newborn Screening, *NR* not reported, *IQ* Total Intelligence Quotient, *SD* standard deviation, *CDI* Children Depression Inventory, *z* z-score, *ADHD* Attention deficit hyperactivity disorder, *NPA* Neuropsychological Assessment, *NI* not investigated

^a^The diet of one patient was relaxed from the age of three years old onward

### Risk of bias assessment

According to the JBI- and the SIGN checklists, only one study was found to be of high quality [19]. Three studies were of low quality, with a high risk of bias [20, 26, 28]. All other studies were found to be of moderate quality. The results of the risk of bias assessment can be found in Additional file 4.

The most frequent issue was the recruitment process of the patients in the eight studies investigating multiple patients. Two studies had a high risk for selection bias due to nonconsecutive- and incomplete inclusion [25], or unclear exclusion criteria for controls and cases [20]. In all five cross-sectional studies the recruitment process was unclear [15, 21–24]. Only one study described the recruitment process in sufficient detail [19]. Moreover, three studies applied exclusion criteria containing neurological or psychiatric disorders [21] and mental retardation [19, 22]. Another major issue was the scarce report of the patients’ age at the initiation of the galactose-restricted diet, making it impossible to infer whether the results might be influenced by late treatment. The presence of psychiatric symptoms was mentioned in three studies but possible effects for, or associations with cognitive outcomes were not tested [15, 19, 21]. Moreover, in the majority of the included studies the association between IQ and performance on the neuropsychological tests was not tested nor accounted for. Finally, there was a large variability between studies in utilized neuropsychological tests. Based on neuropsychological handbooks [8, 16], four studies used tests of moderate psychometric quality and/or with older normative data [15, 21, 23, 26]. One study used a test of unclear psychometric quality [22]. Due to this variability and the moderate to low quality of the included studies, a quantitative meta-analytic approach was not possible. A systematic, narrative approach was utilized for the current review.

### Cognitive outcomes

All results of the neuropsychological tests can be found in Table 2.

**Table 2:** Results of neuropsychological assessment reported in the included studies

Author (year)	Tests	Memory	Information Processing Speed	Attention/Executive functioning	Language	Visuospatial functioning & construction
Antshel et al. (2004) [19]	ROCF BNT WCST BVMI	ROCF IR organization: not impaired (*z* = − 0.57 (*SD* = 0.19); *p* = .307) ROCF IR structural elements accuracy: not impaired (*z* = − 0.25 (*SD* = 0.11); *p* = .338) ROCF IR incidental elements accuracy: impaired (*z* = − 1.47 (*SD* = 0.53); *p <* .001) ROCF DR organization: not impaired (*z* = − 0.58 (*SD* = 0.17); *p* = .387) ROCF DR structural elements accuracy: not impaired (*z* = − 0.63 (*SD* = 0.08); *p* = .316) ROCF DR incidental elements accuracy: impaired (*z* = − 1.43 (*SD* = 0.32); *p <* .001)	NI	WCST completed categories: not impaired (*z* = − 1.32 (*SD* = 0.43); *p* < .001) WCST perseverative errors (%): impaired (*z* = − 1.98 (*SD* = 0.38); *p* < .001)	BNT total correct: not impaired (*z* = − 0.89 (*SD* = 0.18); *p* < .001) BNT total correct with phonemic cue: not impaired (*z* = 0.43 (*SD* = 0.16); *p* = .227)	ROCF Copy organization: not impaired (*z* = − 0.18 (*SD* = 0.10); *p* = .421) ROCF Copy structural elements accuracy: not impaired (*z* = − 0.26 (*SD* = 0.04); *p* = .309) ROCF Copy incidental elements accuracy: not impaired (*z* = − 1.35 (*SD* = 0.45); *p* < .001) BVMI: not impaired (*z* = − 0.77 (*SD* = 0.27); *p* = .089)
Doyle et al. (2010) [21]	Hayling Test BSAT WMS III VOSP - Incomplete letters - Position discrimination	WMS III Auditory IR Index: not impaired (*z* = − 0.53 (*SD* = 1.07), 32.1% < P9) WMS III Auditory DR Index: not impaired (*z* = − 0.37 (*SD* = 1.13), 21.4% < P9) WMS III Auditory Recognition Index: not impaired (*z* = − 0.25 (*SD* = 0.91), 7.1% < P9) WMS III Visual IR Index: not impaired (*z* = − 1.27 (*SD* = 0.73), 39.4% < P9) WMS III Visual DR Index: not impaired (*z* = − 1.18 (*SD* = 0.88), 39.3% < P9)	Hayling Test (*N* = 24): impaired (*z* = − 1.83 (*SD* = 0.4), 29.1% < P9)	WMS III Digit Span (*N* = 27): not impaired (*z* = − 1.03 (*SD* = 0.73), 29.6% < P9) WMS III Spatial Span (*N* = 25): not impaired (*z* = − 0.93 (*SD* = 1.07), 28.0% < P9) WMS III Letter-number sequencing (*N* = 27): not impaired (*z* = − 1.07 (*SD* = 0.87), 29.7% < P9) BSAT (*N* = 24): impaired (*z* = − 1.47 (*SD* = 0.37), 16.7% < P9) Hayling Test (*N* = 24): impaired (*z* = − 1.83 (*SD* = 0.4), 29.1% < P9)	NI	VOSP Incomplete letters (*N* = 23): not impaired (*z* = 0 (*SD* = 1.0), 4.3% < P9) VOSP Position discrimination (*N* = 23): impaired (*z* = − 2.13 (*SD* = 3.38), 43.4% < P9)
Hoffmann et al. (2011) [22]	HWWRT	NI	NI	NI	Impaired: 84.4% of the patients had errors (> cut-off of 1)	NI
Iakovou et al. (2018) [26]	PPVT-III	NI	NI	NI	PPVT-III: not impaired (*z* = 1.20)	NI
Kaufman et al. (1995) [23]	WJ-R COG PPVT-R BVMI	WJ-R Memory for names (*N* = 40): Children: not impaired (*z* = − 0.79 (*SD* = 1.09)), Adults: not impaired (*z* = − 1.33 (*SD* = 0.91))	WJ-R Visual Matching (*N* = 40): Children: impaired (*z* = − 1.86 (*SD* = 1.84)), Adults: not impaired (*z* = − 1.33 (*SD* = 1.27))	WJ-R Analysis-synthesis (*N* = 40): Children: not impaired (*z* = − 1.18 (*SD* = 0.97)), Adults: not impaired (*z* = − 0.99 (*SD* = 1.01)) WJ-R Memory for sentences (*N* = 40): Children: not impaired (*z* = − 1.19 (*SD* = 1.29)), Adults: not impaired (*z* = − 1.21 (*SD* = 0.93))	WJ-R Incomplete words (*N* = 40): Children: not impaired (*z* = − 1.03 (*SD* = 0.96)), Adults: not impaired (*z* = − 0.69 (*SD* = 1.03)) WJ-R Picture vocabulary (*N* = 40): Children: not impaired (*z* = − 1.24 (*SD* = 1.06)), Adults: impaired (*z* = − 1.81 (*SD* = 1.24)) PPVT-R (*N* = 36): Children: not impaired (*z* = − 0.93 (*SD* = 0.89)), Adults: impaired (*z* = − 1.57 (*SD* = 1.09))	WJ-R Visual closure (*N* = 40): Children: not impaired (*z* = − 0.27 (*SD* = 1.0)), Adults: not impaired (*z* = − 0.84 (*SD* = 0.85)) BVMI (*N* = 36): Children: not impaired (*z* = − 1.25 (*SD* = 0.87))
Lewis et al. (2012) [25]	PPVT-4 EVT-2	NI	NI	NI	PPVT-4: Child 2,3,4 impaired (*z* = − 1.67; *z* = − 2.2; *z* = − 2.0), Child 1 not impaired (*z* = 0.73) EVT-2: Child 3,4 impaired (*z* = − 1.93; *z* = − 2.0), Child 1,2 not impaired (*z* = 0.47; *z* = − 1.33)	NI
Lewis et al. (2013) [20]	PLS-4 PPVT-4 EVT-2	NI	NI	NI	PLS-4 Auditory comprehension: not impaired (*z* = − 1.13; *p* ≥ .05) PLS-4 Expressive communication: impaired (*z* = − 1.67; *p* ≥ .05) PPVT-4: not impaired (*z* = − 0.47; *p* ≥ .05) EVT-2: not impaired (*z* = − 1.07; *p* ≥ .05)	NI
Lewis et al. (2013) [27]	PLS-4	NI	NI	NI	PLS-4 Receptive communication: impaired (*z* = − 2.27) PLS-4 Expressive communication: impaired (*z* = − 1.67)	NI
Ng et al. (2003) [28]	PPVT-R	NI	NI	NR	PPVT-R: not impaired (*z* = 1.13)	NR
Van Erven et al. (2017) [24]	ROCF	ROCF IR: impaired (*z* = − 2.0)	NI	NI	NI	NI
Waisbren et al. (2012) [15]	PPVT-4	NI	NI	NI	PPVT-4: not impaired (*z* = − 0.87 (*SD* = 0.98))	NI

*Notes. IR* Immediate Recall, *DR* Delayed Recall, *z* z-score, *SD* standard deviation, *p p*-value, *NI* Not investigated, *NR* Not reported, *P2* Second percentile, *ROCF* Rey-Osterrieth Complex Figure Test, *BNT* Boston Naming Test, *WCST* Wisconsin Card Sorting Test, *BVMI* Beery Developmental test of Visual-Motor Integration, *WRAML* Wide Range Assessment of Memory and Learning, *BSAT* Brixton Spatial Anticipation Test, *WMS III* Wechsler Memory Scale III, *VOSP* Visual Object and Space Perception Battery, *HWWRT* Hierarchische Wortlisten word-repetition test, *PPVT-III* Peabody Picture Vocabulary Test-3, *WJ-R COG* Woodcock-Johnson Psychoeducational Battery - Revised: Tests of cognitive ability, *PPVT-R* Peabody Picture Vocabulary Test – Revised, *PPVT-4* Peabody Picture Vocabulary Test-4, *EVT-2* Expressive Vocabulary Test-2, *PLS-4* Preschool Language Scale-4

#### Information processing speed

Two studies examined information processing speed [21, 23]. A cross-sectional study reporting a total score of two subtests measuring information processing speed and cognitive inhibition, demonstrated an impaired performance averaged across a sample of 24 patients (adults and children), but did not report what process caused the impairment [21]. Thirty percent of the individual patients performed on an impaired level (i.e. 29.1%) in contradiction to about 8% in the normal population [16]. Another cross-sectional study found an impaired visual information processing speed in children (*z* = − 1.86) and a below average result in adults (*z* = − 1.33 [23];).

#### Attention

Two cross-sectional studies addressed attention and found no impairment [21, 23]. However, in both studies the range of performances exceeded the level of impairment, indicating that a proportion of the patients (i.e. 29.6% [21];) performed on an impaired level.

#### Memory

Two studies addressed verbal memory [21, 23]. A cross-sectional study of children and adults found no impairments in verbal information encoding and retrieval [21]. However, 32.1% of the patients performed on an impaired level on encoding and 21.4% on retrieval. The same was found in another cross-sectional study [23].

Three studies examined visual memory [19, 21, 24]. A pediatric case-control study found no impairments on both the immediate- and delayed recall of the structural elements of a complex figure, but the immediate- and delayed recall of incidental elements of the figure was significantly lower in the patient group than in the control group (*p* < .001) and impaired (*z* = − 1.47 and *z* = − 1.43 [19];). A small cross-sectional study reported an impaired overall immediate recall of the same complex figure but did not differentiate the results of the different elements or report the delayed recall-results [24]. Although no memory impairment was found in 28 adults and children in another cross-sectional study, approximately 40% of the patients scored on an impaired level on both immediate- and delayed recall [21].

#### Language

Six studies examined expressive language [19, 20, 22, 23, 25, 27]. No impairment in expressive vocabulary was found in a case-control sample of children although the patient group differed significantly from the control group (*p* < .001). No difference was present if a phonemic cue was presented (*p* = .227 [19];). A cross-sectional study found expressive vocabulary impairment in adults, but not in children [23]. However, the range of performances was large indicating that a proportion of the patients functioned on an impaired level. A case-series reported expressive vocabulary impairment in two out of four children [25]. A poorly designed case-control study found no impairment in another five-year old patient [20]. One cross-sectional study of 32 children and adults found impairments on another aspect of expressive language, namely repetition, measured by a German test of unclear psychometric quality [22]. Lastly, two case studies assessed multiple aspects of expressive language by means of a language scale [20, 27]. Both found an impairment in expressive language.

Seven studies addressed receptive language [15, 20, 23, 25–28]. No receptive vocabulary impairment was found in a cross-sectional study concerning 27 early-treated adults [15]. In contrast, another cross-sectional study concerning both adults and children found impairment in adults, but not in children [23]. However, the scores of patients in both studies showed a large variation indicating that a proportion of the patients performed on an impaired level. A case-series found receptive vocabulary impairment in three children and no impairment in one child [25]. Three case reports, of which one was controlled, found no impairment [20, 26, 28]. A group study reported no impairments in phonological awareness, another basic aspect of receptive language [23]. Lastly, two case studies assessed multiple aspects of receptive language by means of a language scale [20, 27]. Both found an impairment in receptive language.

#### Visuospatial functioning

Two studies addressed visuoconstruction [19, 23]. A case-control study found no impairment in copying a complex figure, but the patients performed significantly worse on copying the incidental parts of the figure in comparison to the controls (*p* < .001 [19];). They also showed no impairment on another copying test, a result also found in another cross-sectional study [23].

Two studies addressed visual perception [21, 23]. Averaged across all 23 patients, impairment in space perception, but not in object perception was found in a cross-sectional study [21]. Only 4.3% of the patients performed on an impaired level on object perception in contradiction to the 43.4% on space perception. Another cross-sectional study found no impairment in object perception, but scores differed extensively between individual patients [23].

#### Executive functioning

Three studies examined executive functioning [19, 21, 23], which is an umbrella term for several higher-order functions of which four were investigated in patients with CG (i.e. working memory, abstract thinking, cognitive flexibility and cognitive inhibition). A cross-sectional study found no working memory impairment after averaging across all patients, but 30% of the patients performed on an impaired level [21]. A pediatric case-control study assessed abstract thinking together with cognitive flexibility [19]. The patients performed worse than controls on both abstract thinking and cognitive flexibility (*p* < .001), but only the performance on cognitive flexibility reached a level of impairment (*z* = − 1.98) indicating impairment in cognitive flexibility alone. Another cross-sectional study also utilized a test measuring both abstract thinking and cognitive flexibility [21]. They found an impaired performance averaged across all patients, and a proportion of 16.7% of the patients performing on an impaired level. However, separate scores for abstract thinking and cognitive flexibility were not reported, leaving it unclear whether the low performance was due to cognitive flexibility impairment alone. A cross-sectional study found scores within normal limits on an abstract thinking test which does not involve cognitive flexibility, however large differences between patients were present [23]. One cross-sectional study reported impairments on a test measuring cognitive inhibition [21]. However, this result was based on two tests measuring information processing speed and cognitive inhibition. Therefore, it is unclear which process underlies the impaired performance.

#### Social cognition

Since none of the included studies examined social cognition with standardized neuropsychological tests, it remains unclear whether patients with CG are impaired in social cognition.

## Discussion

The current systematic review examined the incidence of cognitive impairment in patients with CG and reviewed the impairment in specific cognitive domains. Eleven studies were identified, including three case reports and one case-series. Of the eight studies investigating multiple patients, the quality was in seven studies moderate to low. Moreover, the number of studies per cognitive domain was low.

The review revealed that large differences exist amongst patients with CG. The averaged performance of the patients reported in each group study was often on a below average to low level, while a proportion of the patients performed on an impaired level. Twenty to 40 % of the patients performed on an impaired level on attention and memory, and, according to one study, on working memory. The range of vocabulary performances of the individual patients also exceeded the level of impairment, however specific percentages of the proportion of patients performing on an impaired level remained unknown. Evidence for impairments in other aspects of language functioning was mainly limited to case studies. The average level of performance did reach an impaired level for information processing speed, space perception, cognitive flexibility and cognitive inhibition, but the evidence was based on only a small number of studies. There is some indication that abstract thinking and visuoconstruction are relatively spared. Social cognition was not investigated at all. These results suggest that specific cognitive impairments indeed underlie the lower level of intellectual functioning. However, a specific cognitive profile cannot be determined due to individual differences between patients and limited number of merely small studies. A large number of studies investigating cognition in CG was excluded in this review since they only utilized developmental screening- or intelligence batteries. This represents the initial main focus on clinically assessing developmental delay and intelligence only in patients with CG.

To improve the knowledge concerning cognitive functioning in CG, well-designed and well-reported studies covering multiple cognitive domains should be performed. The risk of selection bias needs to be lowered for example by describing the recruitment process in more detail and by refraining from the exclusion of patients with other long-term complications of CG (i.e. mental retardation and neurological or psychiatric disorders). Excluding these patients will lead to an underestimation of cognitive impairments that patients with CG may encounter. However, the inclusion of these patients will also add confounding factors which will need to be taken into account in the statistical analysis. Moreover, the influence of late treatment on cognitive development [11, 12] needs to be acknowledged by either solely including early-treated patients (i.e. < 8 weeks) or preferably distinguishing the results of early- and late treated patients. If available, the pathogenic mutations carried by the patients should be reported as well in order to establish the presence of patients with the S135 L variation which is associated with a better clinical outcome [13]. Possible confounders such as anxiety and depression [15] should be considered in the analysis of cognitive results, since they can influence cognitive performance [14] just as the level of intelligence [8]. Future neuropsychological assessments need to cover several cognitive domains with preferably multiple tests per domain. Important domains include information processing speed, attention, memory, language, visuospatial perception, executive functioning and social cognition. Information processing speed and executive functioning are especially important to be investigated in light of brain imaging findings in patients with CG. First, white matter abnormalities were found [29, 30] which are associated with lower information processing speed in both healthy- and patient populations [31]. Second, grey matter abnormalities were found within areas involved in executive functioning (i.e. medial prefrontal cortex and inferior frontal gyrus [32] and the orbital frontal lobes [33]). Finally, the report of cognitive results needs to involve the quantitative results of tests, including subtests. The mean raw score plus standard deviation and interquartile range, and normative score need to be reported as well as the proportion of patients performing on an impaired level to acknowledge the large individual differences between patients. Unfortunately, an important limitation of studying rare diseases is the absence of large sample sizes. In order to understand these differences in cognitive functioning between CG-patients, large patient cohorts are necessary. Initiatives in which data of multiple patient cohorts are combined (e.g. the recently developed Galactosemia Patient Registry [34]) might help to facilitate studies investigating the relation between different disease parameters (e.g. variations in the *GALT* gene) and the severity of cognitive impairment in a larger group of patients. This will also make it possible to examine the association of cognition and other long-term complications of classical galactosemia such as movement disorders (e.g. tremor, ataxia, dystonia). Lower intellectual functioning has been found to be more frequent in patients with motor dysfunction [35], raising the question whether cognitive impairment in patients with CG is associated with other long-term complications of CG. Therefore, cognitive functioning should be part of this registry as well.

The recent international clinical guideline for the management of classical galactosemia [3] acknowledges that certain cognitive domains (i.e. executive functioning, information processing speed and visual spatial comprehension) need to be clinically assessed, additionally to the routine assessment of general mental abilities utilizing intelligence tests. This review supports this recommendation, but also highlights that neuropsychological assessment of CG-patients should not be limited to these three cognitive domains. Preferably all cognitive domains should be assessed by means of a neuropsychological assessment. In this way patients and their caregivers will gain more insight in the patients’ cognitive strengths and weaknesses. This will result in a better understanding by the patient, caregivers, teachers and coworkers and consequently a more suitable guidance plan can be made and access to appropriate interventions (e.g. compensatory strategy training [36]) can be provided. Ultimately, this could improve the health-related quality of life which is affected by the lower level of cognitive functioning [5].

### Methodological limitations

The review might have suffered from selection bias due to the inclusion of English, original articles only, and the exclusion of studies in which the diagnosis CG remained unclear or a specific sample of CG-patients was drawn based on clinical outcome. This could have eliminated studies investigating cognitive functioning with standardized tests. However, only seven studies were excluded because of the latter two reasons, keeping the current sample of patients with CG representative of the entire CG population. Strengths of the current review include an extensive search strategy to incorporate all studies related to the long-term outcome of CG, and the inclusion of studies reporting quantitative data of standardized neuropsychological tests only.

## Conclusions

This systematic review revealed that a large proportion of the patients (i.e. 20–40%) seems to perform on an impaired level on attention, memory and/or vocabulary. Evidence for impairments in information processing speed, language, visuospatial functioning, working memory, cognitive flexibility and cognitive inhibition was limited due to the small number of studies investigating these cognitive functions. Social cognition was not examined at all. Both clinicians and researchers encountering patients with CG need to be aware of possible cognitive impairments in different degrees of severity. However, they need to be conscious that only tentative conclusions regarding cognitive impairment can be drawn based on the current scientific evidence. All results need to be evaluated in larger, well-designed studies specifying the cognitive functioning and individual differences between CG-patients in order to make a reliable judgement. This can be the basis for the development of intervention strategies.

## Supplementary information

**Additional file 1.** PRISMA checklist. Completed PRISMA checklist for the current systematic review.

**Additional file 2.** Clusters of related articles identified in VOSviewer. Visualization of the identified clusters of related articles in VOSviewer which were used to manually omit irrelevant clusters of articles in the search strategies in EMBASE and MEDLINE (see Additional file 3).

**Additional file 3.** Search strategies. Search strategies for MEDLINE, EMBASE and PsychINFO used in the current systematic review.

**Additional file 4.** Risk of bias assessment. The results of the risk of bias assessment according to the Scottish Intercollegiate Guidelines Network (SIGN) quality appraisal checklists and the Joanna Briggs Institute (JBI) critical appraisal checklists.

## Data Availability

The datasets used and/or analysed during the current study are available from the corresponding author on reasonable request.

## Abbreviations

CG: Classical galactosemia
GALT: Galactose-1-phosphate uridylyltransferase
IQ: Intelligence quotient
JBI: Joanna Briggs Institute
PRISMA: Preferred Reporting Items for Systematic Reviews and Meta-Analyses
SIGN: Scottish Intercollegiate Guidelines Network
