# Supplementary figures and images for: Cognitive functioning in patients with classical galactosemia: a systematic review
Source: Orphanet J Rare Dis. 2019 Oct 18;14:226. doi: 10.1186/s13023-019-1215-1 (PMC6798502; doi:10.1186/s13023-019-1215-1)

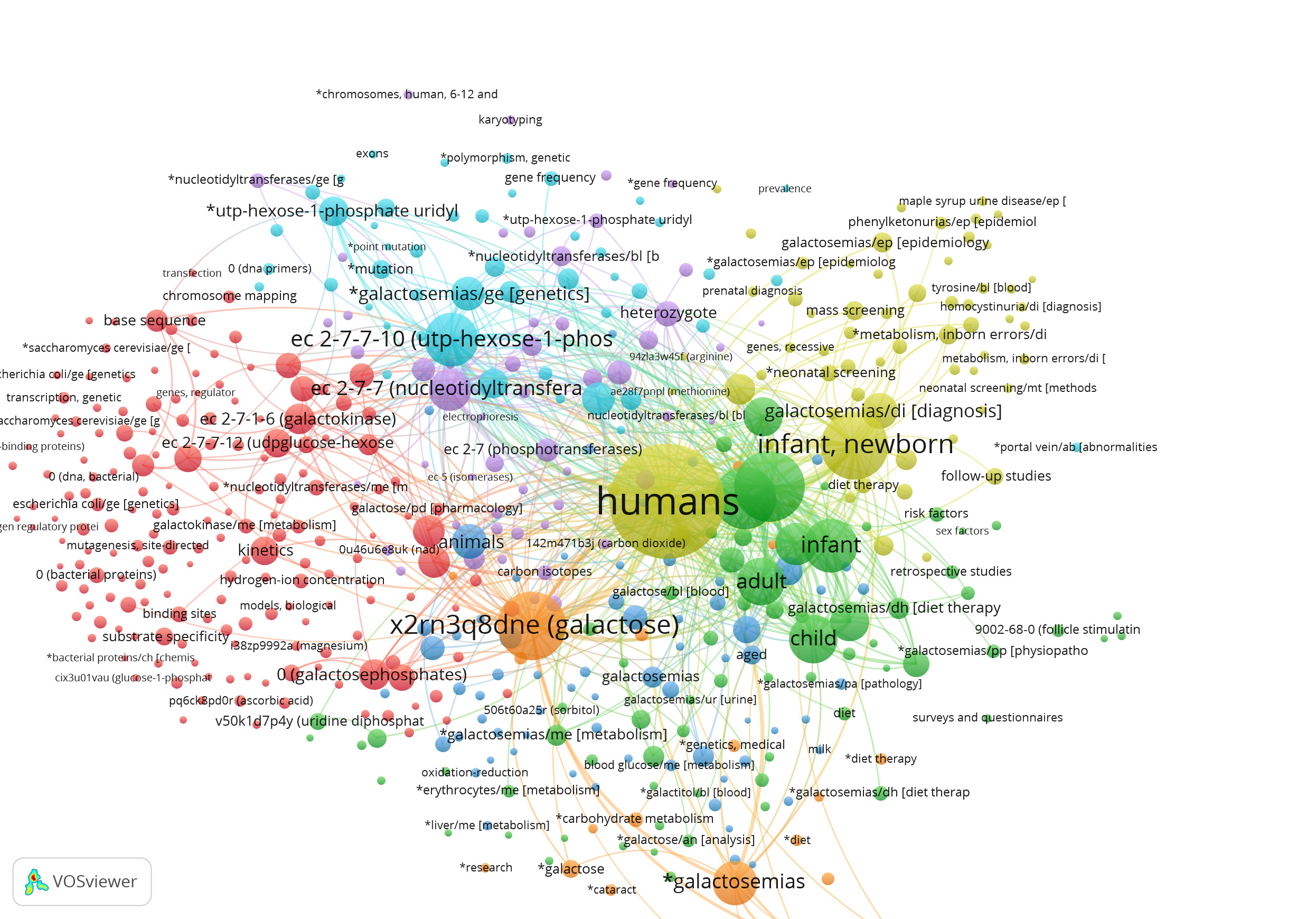

Supplement: Supplementary file 2 — Additional file 2. Clusters of related articles identified in VOSviewer. Visualization of the identified clusters of related articles in VOSviewer which were used to manually omit irrelevant clusters of articles in the search strategies in EMBASE and MEDLINE (see Additional file 3). [file 13023_2019_1215_MOESM2_ESM.jpeg]
