# Supplementary material for: Cognitive functioning in patients with classical galactosemia: a systematic review
Source: Orphanet J Rare Dis. 2019 Oct 18;14:226. doi: 10.1186/s13023-019-1215-1 (PMC6798502; doi:10.1186/s13023-019-1215-1)
Supplement: Supplementary file 3 — Additional file 3. Search strategies. Search strategies for MEDLINE, EMBASE and PsychINFO used in the current systematic review. [file 13023_2019_1215_MOESM3_ESM.docx]

Table 1

*Search strategy MEDLINE (Ovid MEDLINE(R) Epub Ahead of Print, In-Process & Other Non-Indexed Citations, Ovid MEDLINE(R) Daily and Ovid MEDLINE(R) <1946 to Present>)*

| # | Search terms |
| --- | --- |
| 1 | galactosemias/ or UTP-Hexose-1-Phosphate Uridylyltransferase/ or UDPglucose-Hexose-1-Phosphate Uridylyltransferase/ |
| 2 | (galactos?emia? or GALT deficien* or (galactose adj2 phosphate) or "utp hexose 1 phosphate" or "UDPglucose-Hexose-1-Phosphate").ab,kf,ti. |
| 3 | ("EC 2-7-7-10" or "EC 2-7-7-12" or "9016-11-9" or "9026-21-5").rn. |
| 4 | NTR2869.ab. |
| 5 | or/1-4 |
| 6 | animals/ not humans/ |
| 7 | (animal? or rat? or mouse or mice or rodent?).kf,ti. |
| 8 | 6 or 7 |
| 9 | 5 not 8 |
| 10 | (kinetics or base sequence or galactokinase or bacterial proteins or protein confirmation or crystallography or genetic transcription or chromosome mapping or udb glucose 4-epime).ab,kf,ti. |
| 11 | "ec 2.7.1.6".rn. |
| 12 | "ec 5.1.3.2".rn. |
| 13 | or/10-12 |
| 14 | review.ab,kf,pt,ti. |
| 15 | 13 not 14 [notting out VOS red] |
| 16 | (dna mutation analysis or polymerase chain reaction or exons or gene frequency).ab,kf,ti. [notting out VOS blue] |
| 17 | (cultured cells or clincal enzyme test* or nucleotidyltransfera* or phosphotransfera*).ab,kf,ti. |
| 18 | "ec 2.7.7".rn. |
| 19 | "ec 2.7".rn. |
| 20 | or/17-19 [notting out VOS purple] |
| 21 | 9 not (15 or 16 or 20) |
| 22 | remove duplicates from 21 |

Table 2

*Search strategy EMBASE (Ovid Embase Classic+Embase 1947 to 2018 October 22)*

| # |  | Search terms |
| --- | --- | --- |
| 1 |  | galactose 1 phosphate uridylyltransferase/ or hexose 1 phosphate uridylyltransferase/ or galactosemia/ |
| 2 |  | (galactos?emia? or GALT deficien* or (galactose adj2 phosphate) or "utp hexose 1 phosphate" or "UDPglucose-Hexose-1-Phosphate").ab,kw,ti. |
| 3 |  | ("EC 2.7.7.10" or "EC 2.7.7.12").ab,ez. [EC numbers] |
| 4 |  | ("9016-11-9" or "9026-21-5").rn. [CAS numbers] |
| 5 |  | NTR2869.ab,cn. |
| 6 |  | or/1-5 |
| 7 |  | (animal/ or animal experiment/ or animal model/ or nonhuman/) not human/ |
| 8 |  | (animal? or rat? or mouse or mice or rodent?).kw,ti. |
| 9 |  | 7 or 8 |
| 10 |  | 6 not 9 |
| 11 |  | (kinetics or base sequence or galactokinase or bacterial proteins or protein confirmation or crystallography or genetic transcription or chromosome mapping or udb glucose 4-epime).ab,kw,ti. |
| 12 |  | "ec 2.7.1.6".ab,ez. |
| 13 |  | "ec 5.1.3.2".ab,ez. |
| 14 |  | or/11-13 |
| 15 |  | review.ab,kw,pt,ti. |
| 16 |  | 14 not 15 [notting out VOS red] |
| 17 |  | (dna mutation analysis or polymerase chain reaction or exons or gene frequency).ab,kw,ti. [notting out VOS blue] |
| 18 |  | (cultured cells or clincal enzyme test* or nucleotidyltransfera* or phosphotransfera*).ab,kw,ti. |
| 19 |  | "ec 2.7.7".ab,ez. |
| 20 |  | "ec 2.7".ab,ez. |
| 21 |  | or/18-20 [notting out VOS purple] |
| 22 |  | 10 not (16 or 17 or 21) |
| 23 |  | remove duplicates from 22 |

Table 3

*Search strategy PsychINFO (Ovid PsycINFO <1806 to October Week 3 2018>)*

| # | Search terms |
| --- | --- |
| 1 | (galactos?emia? or GALT deficien* or (galactose adj2 phosphate) or "utp hexose 1 phosphate" or "UDPglucose-Hexose-1-Phosphate").ab,id,ti. |
