# Supplementary material for: Cognitive functioning in patients with classical galactosemia: a systematic review
Source: Orphanet J Rare Dis. 2019 Oct 18;14:226. doi: 10.1186/s13023-019-1215-1 (PMC6798502; doi:10.1186/s13023-019-1215-1)
Supplement: Supplementary file 4 — Additional file 4. Risk of bias assessment. The results of the risk of bias assessment according to the Scottish Intercollegiate Guidelines Network (SIGN) quality appraisal checklists and the Joanna Briggs Institute (JBI) critical appraisal checklists. [file 13023_2019_1215_MOESM4_ESM.docx]

Table 1

*Risk of bias assessment for case-control studies based on the SIGN checklist for case-control studies*

| Author (year) | Research question | Comparability population of cases and controls | Exclusion criteria | Participation rate | Similarity participants and non-participants | Clear definition of cases | Clear definition of controls | Blinding | Outcome assessment | Confounding | Report of results |
| --- | --- | --- | --- | --- | --- | --- | --- | --- | --- | --- | --- |
| Antshel et al. (2004)(1) | Low | Low | Low | Low | ? | Low | Low | ? | Low | Low | Low |
| Lewis et al. (2013)(2) | Low | ? | ? | ? | High | Low | Low | ? | Low | High | Low |
| *Notes*. Low = Low risk of bias. High = High risk of bias. ? = Unclear. | | | | | | | | | | | |

Table 2

*Risk of bias assessment for cross-sectional studies based on the JBI critical appraisal checklist for prevalence studies*

| Author (year) | Sample frame | Sampling procedure | Sample size | Description subjects and setting | Validity of methods for identification of disorder and condition | Outcome assessment | Response rate |
| --- | --- | --- | --- | --- | --- | --- | --- |
| Doyle et al. (2010)(3) | Low | ? | Low | Low | Low | Low | ? |
| Hoffmann et al. (2011)(4) | Low | ? | Low | Low | ? | Low | High |
| Kaufman et al. (1995)(5) | Low | ? | Low | Low | Low | High | ? |
| Van Erven et al. (2017)(6) | Low | ? | High | Low | Low | ? | ? |
| Waisbren et al. (2012)(7) | Low | ? | Low | Low | Low | ? | ? |
| *Notes*. Low = Low risk of bias. High = High risk of bias. ? = Unclear. | | | | | | | |

Table 3

*Risk of bias assessment for case series based on the JBI critical appraisal checklist for case series*

| Author (year) | Inclusion criteria | Measurement of condition | Validity of methods for identification of disorder | Consecutive inclusion | Complete inclusion | Description of demographics | Description of clinical information | Description of outcomes | Description of site/clinic |
| --- | --- | --- | --- | --- | --- | --- | --- | --- | --- |
| Lewis et al. (2012)(8) | Low | Low | Low | High | High | Low | Low | Low | Low |
| *Notes*. Low = Low risk of bias. High = High risk of bias. | | | | | | | | | |

Table 4

*Risk of bias assessment for case reports based on the JBI critical appraisal checklist for case reports*

| Author (year) | Description of patients’ demographic characteristics | Description of patients’ history | Description of current clinical condition | Description of diagnostic tests, assessment methods and results | Take-away lessons |
| --- | --- | --- | --- | --- | --- |
| Iakovou et al.  (2018)(9) | Low | High | High | Low | Low |
| Lewis et al. (2013)(10) | Low | Low | High | Low | Low |
| Ng et al.  (2003)(11) | Low | High | High | High | Low |
| *Notes*. Low = Low risk of bias. High = High risk of bias. | | | | | |

**References**

1. Antshel KM, Epstein IO, Waisbren SE. Cognitive strengths and weaknesses in children and adolescents homozygous for the galactosemia Q188R mutation: a descriptive study. Neuropsychology. 2004;18(4):658-64.

2. Lewis FM, Coman DJ, Syrmis M, Kilcoyne S, Murdoch BE. Impaired language abilities and pre-linguistic communication skills in a child with a diagnosis of galactosaemia. Early Child Dev. Care. 2013;183(12):1747-57.

3. Doyle CM, Channon S, Orlowska D, Lee PJ. The neuropsychological profile of galactosaemia. J Inherit Metab Dis. 2010;33(5):603-9.

4. Hoffmann B, Wendel U, Schweitzer-Krantz S. Cross-sectional analysis of speech and cognitive performance in 32 patients with classic galactosemia. J Inherit Metab Dis. 2011;34(2):421-7.

5. Kaufman FR, McBride-Chang C, Manis FR, Wolff JA, Nelson MD. Cognitive functioning, neurologic status and brain imaging in classical galactosemia. Eur J Pediatr. 1995;154(2):S2-S5.

6. Van Erven B, Jansma BM, Rubio-Gozalbo ME, Timmers I. Exploration of the brain in rest: resting-state functional MRI abnormalities in patients with classic galactosemia. Sci Rep. 2017;7(1):9095.

7. Waisbren SE, Potter NL, Gordon CM, Green RC, Greenstein P, Gubbels CS, et al. The adult galactosemic phenotype. J Inherit Metab Dis. 2012;35(2):279-86.

8. Lewis FM, Coman DJ, Syrmis M, Kilcoyne S, Murdoch BE. Differential phonological awareness skills in children with classic galactosemia: A descriptive study of four cases. JIMD Rep. 2012;10:45-52.

9. Iakovou K, Kalogerakou M, Schulpis K. A patient with classical galactosemia is graduated with a university degree. J Pediatr Endocrinol Metab. 2018;31(10):1147-8.

10. Lewis FM, Coman DJ, Syrmis M, Kilcoyne S, Murdoch BE. Charting a seven-year trajectory of language outcomes for a child with galactosemia. J Dev Behav Pediatr. 2013;34(6):414-8.

11. Ng W, Xu Y, Wong L, Kaufman F, Buist N, Donnell G. Two adult galactosaemia females with normal ovarian function and identical GALT mutations (Q188R/R333G). J Inherit Metab Dis. 2003;26(1):75-9.
